# Non-invasive imaging techniques for early diagnosis of bilateral cardiac dysfunction in pulmonary hypertension: current crests, future peaks

**DOI:** 10.3389/fcvm.2024.1393580

**Published:** 2024-05-09

**Authors:** Ashfaq Ahmad, Yifan Zou, Peng Zhang, Lingling Li, Xiaoyu Wang, Yousen Wang, Fenling Fan

**Affiliations:** ^1^ Department of Cardiovascular Medicine, First Affiliated Hospital, Xi'an Jiaotong University, Xi'an, Shaanxi, China; ^2^ School of Economics and Finance, Xi'an Jiaotong University, Xi'an, Shaanxi, China

**Keywords:** pulmonary hypertension, right and left ventricular interdependence, ventricular dysfunction, cardiac magnetic resonance, echocardiography

## Abstract

Pulmonary arterial hypertension (PAH) is a chronic and progressive disease that eventually leads to heart failure (HF) and subsequent fatality if left untreated. Right ventricular (RV) function has proven prognostic values in patients with a variety of heart diseases including PAH. PAH is predominantly a right heart disease; however, given the nature of the continuous circulatory system and the presence of shared septum and pericardial constraints, the interdependence of the right and left ventricles is a factor that requires consideration. Accurate and timely assessment of ventricular function is very important in the management of patients with PAH for disease outcomes and prognosis. Non-invasive modalities such as cardiac magnetic resonance (CMR) and echocardiography (two-dimensional and three-dimensional), and nuclear medicine, positron emission tomography (PET) play a crucial role in the assessment of ventricular function and disease prognosis. Each modality has its own strengths and limitations, hence this review article sheds light on (i) ventricular dysfunction in patients with PAH and RV–LV interdependence in such patients, (ii) the strengths and limitations of all available modalities and parameters for the early assessment of ventricular function, as well as their prognostic value, and (iii) lastly, the challenges faced and the potential future advancement in these modalities for accurate and early diagnosis of ventricular function in PAH.

## Introduction

Pulmonary arterial hypertension (PAH) stands as a chronic and progressive ailment culminating in right heart failure (HF) and eventual fatality within a relatively brief timeframe if left unattended. A distinct manifestation of severe precapillary pulmonary hypertension (PH), an advancing pulmonary vascular disorder culminating in right HF and demise, is chronic thromboembolic pulmonary hypertension (CTEPH), acknowledged as a complication arising from pulmonary embolism (PE). These two variations of precapillary PH may exhibit analogous pathological alterations within the distal pulmonary vasculature, encompassing intimal thickening and remodeling of pulmonary resistance vessels, eccentric intimal fibrosis, intimal fibromuscular proliferation, and the development of plexiform lesions (1). PH is one of the most common diseases, often associated with other underlying conditions (i.e., Group 2 and 3 PH). Conversely, pulmonary vascular resistance (PVR) is not forcibly increased in Group 2 PH, considering that the isolated postcapillary form is, by definition, characterized by PVR < 2 units (2). PH precipitates a multifaceted cascade of pathological events within the right ventricle (RV), leading to hypertrophy, dilatation, fibrosis, and metabolic perturbations. This intricate process is instigated by the heightened afterload imposed on the RV, ultimately culminating in the manifestation of right heart failure and consequential mortality (3). Prior investigations have substantiated the pivotal role of RV function as a significant prognostic determinant in patients afflicted by PH, irrespective of the diverse etiological factors underlying the condition (4). Given the continuous nature of the circulatory system and the holistic nature of the left and right ventricles, which are determined by the presence of a shared septum and pericardial constraints, the interdependence of the RV and left ventricles (LV) is a factor that requires consideration (5). Although the overwhelming majority of studies have emphasized RV dysfunction in individuals with PAH, a recent study indicated that LV dysfunction may also be present in this population (6). Hence, accurate and early diagnosis of PAH, as well as cardiac function, plays a crucial role in the treatment of such patients.

Right heart catheterization (RHC) is known to be a gold standard modality for the diagnosis and treatment strategies of PAH (7). According to the guidelines issued by the Sixth World Symposium on Pulmonary Hypertension (WSPH) in 2018, hemodynamic diagnosis via RHC, with a mean pulmonary artery pressure (mPAP) above 20 mmHg indicating PAH updated from the previous 25 mmHg, delineates PAH into five distinct groups (1). The WSPH classifications of PH are presented in Table 1. Hemodynamic changes in the RV, including parameters like mPAP, PVR, and a diminished RV cardiac index (CI), exhibit significant associations with unfavorable clinical outcomes in PAH as identified through RHC (8). The non-invasive approach toward early diagnosis of ventricular dysfunction in daily practice has its own strengths and limitations. The aim of this literature review is to briefly illuminate the current status of PH and elucidate the following: (i) the intricacies of ventricular dysfunction and ventricular interdependence observed in patients with PAH, (ii) the strengths and limitations of non-invasive imaging modalities utilized for the early detection of ventricular dysfunction in these individuals, and (iii) subsequently the challenges faced and possible potential future directions in these modalities for the management of such patients.

**Table 1:** The Sixth WSPH classification of PAH (1).

PAH 1.1. Idiopathic PAH 1.2. Heritable PAH 1.3. Drug- and toxin-induced PAH 1.3.1 Definite *Aminorex* *Fenfluramine* *Dexfenfluramine* *Benfluorex* *Methamphetamines* *Dasatinib* *Toxic rapeseed oil* 1.3.2 Possible *Cocaine* *Phenylpropanolamine* *L-tryptophan* *St John's wort* *Amphetamines* *Interferon-*α *and -β* *Alkylating agents* *Bosutinib* *Direct-acting antiviral agents against hepatitis C virus Leflunomide* *Indirubin (Chinese herb Qing-Dai)* 1.4. PAH associated with: 1.4.1 Connective tissue disease 1.4.2 HIV infection 1.4.3 Portal hypertension 1.4.4 Congenital heart disease 1.4.5 Schistosomiasis 1.5. PAH long-term responders to calcium channel blockers 1.5.1 Acute pulmonary vasoreactivity^a^ for patients with idiopathic, hereditable, or drug-induced PAH (reduction of mPAP ⩾ 10 mmHg to reach an absolute value of mPAP ⩽ 40 mmHg increased or unchanged cardiac output). 1.5.2 Long-term response to CCBs [NYHA Functional Class I/II With sustained hemodynamic improvement (same or better than achieved in the acute test) after at least 1 year on CCBs only]. 1.6. PAH with overt features of venous/capillaries (PVOD/PCH) involvement.^b^ 1.6.1 Pulmonary function tests (decreased DLCO, frequently <50% severe hypoxia). 1.6.2 Chest HRCT (septal lines, centrilobular ground-glass opacities/nodules, mediastinal lymph node enlargement). 1.6.3 Response to PAH therapy (possible pulmonary edema) 1.6.4 Genetic background (biallelic EIF2AK4 mutations) 1.6.5 Occupational exposure (organic solvent i.e., trichloroethylene) 1.7. Persistent PH of the newborn syndrome PH due to left heart disease 1.8. PH due to heart failure with preserved LVEF 1.9. PH due to heart failure with reduced LVEF 1.10. Valvular heart disease 1.11. Congenital/acquired cardiovascular conditions leading to postcapillary PH PH due to lung diseases and/or hypoxia 1.12. Obstructive lung disease 1.13. Restrictive lung disease 1.14. Other lung disease with mixed restrictive/obstructive pattern 1.15. Hypoxia without lung disease 3.5 Developmental lung disorders PH due to pulmonary artery obstructions 1.16. Chronic thromboembolic PH 1.17. Other pulmonary artery obstructions 1.17.1 Sarcoma (high or intermediate grade) or angiosarcoma 1.17.2 Other malignant tumors *Renal carcinoma* *Uterine carcinoma* *Germ cell tumors of the testis* *Other tumors* 1.17.3 Non-malignant tumors *Uterine leiomyoma* 1.17.4 Arteritis without connective tissue disease 1.17.5 Congenital pulmonary artery stenoses 1.17.6 Parasites Hydatidosis PH with unclear and/or multifactorial mechanisms 1.18. Haematological disorders * Chronic hemolytic anemia* *Myeloproliferative disorders* 1.19. Systemic and metabolic disorders *Pulmonary Langerhans cell histiocytosis* *Gaucher disease* *Glycogen storage disease* *Neurofibromatosis* *Sarcoidosis* 1.20. Others Chronic renal failure with or without hemodialysis Fibrosing mediastinitis 1.21. Complex congenital heart disease (9) 1.21.1 Segmental pulmonary hypertension *Isolated pulmonary artery of ductal origin* *Absent pulmonary artery* *Pulmonary atresia with ventricular septal defect and major aortopulmonary collateral arteries* *Hemitruncus* *Other* 1.21.2 Single ventricle *Unoperated* *Operated* 1.21.3 Scimitar syndrome

^a^ Nitric oxide (10–20 ppm) is recommended for performing vasoreactivity testing, but i.v. epoprostenol, i.v. adenosine or inhaled iloprost can be used as alternatives.

^b^ Signs evocative of venous and capillary (pulmonary veno-occlusive disease/pulmonary capillary hemangiomatosis) involvement.

CCB, calcium channel blocker; DLCO, diffusing capacity of the lung for carbon monoxide; HRCT, high-resolution computed tomography; LVEF, left ventricular ejection fraction; NYHA; New York Heart Association; PAH, pulmonary arterial hypertension; PH, Pulmonary hypertension; PVOD, pulmonary veno-occlusive disease; PCH, pulmonary capillary hemangiomatosis.

## Ventricular dysfunction in patients with PAH

### RV dysfunction in patients with PAH

Although changes in the pulmonary vasculature are the primary cause of PAH severity of symptoms, and survival is strongly associated with right ventricular function, right heart failure is the main cause of mortality in patients with PAH (10). The pathophysiological mechanisms underlying PAH, although not fully elucidated, encompass a myriad of intricate processes including vasoconstriction, inflammatory responses, thrombotic events, and abnormal endovascular remodeling. Although our understanding remains incomplete, these multifaceted pathways contribute to the complex nature of PAH (11). The eventual outcome manifests as a gradual constriction of the pulmonary arteries, accompanied by elevated PAP and resistance. In response to the pressure overload, the right heart initially adapts through concentric hypertrophy; however, over time the RV experiences significant dilation leading to complications such as tricuspid regurgitation as well as volume and pressure overload. This progressive RV dilation causes a decline in RV contractility. Concurrently, the leftward bowing of the septum hampers left ventricular filling, resulting in decreased LV stroke volume (SV) and cardiac output. The diminished cardiac output, coupled with heightened RV intramural pressure, compromises systolic RV coronary perfusion, which normally occurs throughout the entire cardiac cycle encompassing both the systole and diastole. Consequently, a detrimental cycle ensues characterized by RV dysfunction and low cardiac output, ultimately culminating in progressive RV failure and eventual mortality (10, 12).

Regardless of etiology, RV adaptation to increased pressure load is the main determinant of outcome (13, 14); however, mortality because of RV failure is significantly higher in systemic sclerosis-related PAH (SSc-PAH) than idiopathic PAH (IPAH) (15), and the studies also suggest that patients with SSc-PAH have depressed RV contractility compared with associated PAH (APAH) at similar afterloads may be because of intrinsic systolic function rather than enhanced pulmonary vascular resistive and pulsatile loading (16).

### LV dysfunction in patients with PAH

Although PAH is predominantly a right heart disease and RV dysfunction has been the focus of research on people with PAH, a recent study suggested that LV dysfunction may also exist in such a population (6, 17). The deleterious consequences arising from RV dysfunction, dilatation, and hypertrophy on left ventricular function commonly referred to as interventricular interaction, have been observed in patients with pulmonary hypertension (PH) (18). Manifestations such as septal flattening and leftward deviation, characteristic of a “D-shaped” left ventricle, ensue due to heightened RV pressure, and these geometric alterations may exert an adverse impact on LV function (19). Furthermore, the compromised RV stroke volume, resulting in an under filled left ventricle, has the potential to detrimentally influence longitudinal LV function. Interestingly, pulmonary acceleration time (PAcT) and TR Doppler velocities (reflecting RV systolic pressures) correlated with early diastolic (E) velocities at the septal and mitral valve levels. These findings suggest that PH had a greater impact on the diastolic compared with the systolic cycle of the LV (20). Echocardiography reveals classic RV alterations in severe secondary pulmonary arterial hypertension (sPAH), including RV dilatation, decreased contractility, and paradoxical motion of the interventricular septum (IVS) with concomitant right heart failure.

However, a notable scarcity of data exists regarding the impacts of RV failure on the left heart chambers, specifically in light of the potential geometric changes in the RV due to pressure overload in PAH, which may adversely affect the LV (21). It has been shown that LV dysfunction is associated with a worse prognosis and more severe disease and may develop as a subsequent result of primary PH (22). Left ventricular global longitudinal strain (LVGLS) was found to be independently linked with mortality in a recent study involving individuals with severe PAH (19). In a study, 114 patients with severe pulmonary hypertension with normal LV ejection fraction were compared with 70 normal controls of similar age and gender distribution. The authors found that the LVGLSs were found reduced in patients with severe PH. LVGLS [HR = 1.11 (CI, 1.01–1.22); *P* = .04] was found to be independently associated with mortality (19). Similarly, another concept proposed is that RV pressure overload eventually leads to LV diastolic dysfunction in PH (23). Pathophysiologically, LV diastolic dysfunction is due to a distortion of IVS toward the LV in the presence of RV pressure overload (24).

### RV–LV interdependence in PAH

Although PAH is defined by normal LV filling pressures, LV performance may be compromised. Theoretically, patients with PAH should have normal left cardiac structure and function. Nevertheless, because they share an IVS and pericardial sac, the RV and LV do not operate independently (25). Since the IVS is shared by both ventricles within the same pericardial sac, increasing RV pressure load causes the septum to bow toward the left ventricle, which alters LV geometry and reduces LV filling (26). The majority of patients with PAH have right-sided disease, while in later stages of the condition reduced left-sided function may also become apparent (6). The interdependence of the RV and LV is an issue that needs to be taken into consideration because of the continuous structure of the circulatory system and the holistic character of the left and right ventricles, which is defined by the presence of a shared septum and pericardial limitations (5). From the perspective of the cardiac systolic–diastolic phase, prior research has shown that increased RV afterload, particularly in people with PAH, causes an elongated systolic phase, which in turn shortens diastole and influences the LV diastolic function (27). A study by Gan et al. (26) aimed to investigate the contribution of direct right-to-left ventricular interaction to LV filling and SV in 46 patients with PAH by using cardiac magnetic resonance (CMR). When comparing patients with PAH to control subjects, there was a decrease in SV, left ventricular end-diastolic volume (LVEDV), and LV peak filling rate (26). Their findings indicate that, in patients with PAH, ventricular interaction mediated by IVS compromises LV filling. As a result, aberrant RV loading reduces the RV cardiac output by influencing the ventricle's function through both direct and indirect interactions. We may examine some of the results more closely if we keep this idea of ventricular interdependence in mind. First, it is intriguing to note that patients with PAH have lower LV septal circumferential strain (CS) than controls. In a typical situation, the LV pressure is higher than the RV. Like the rest of the LV, the IVS is circumferentially tensioned to endure this pressure gradient (28). It is interesting to note that LV CS did not decrease in patients with concurrent pulmonary hypertension and aortic stenosis (29). The main factor for lower LV septal CS in PAH appears to be RV pressure load in conjunction with septal displacement (29). Takamura et al. (30) also reported an intriguing finding: improvements in LV global, septal, and non-septal CS were linked to improvements in hemodynamic measures. Increased LV CS was more closely linked to this improvement than increased RV free wall longitudinal strain (FWLS) (30). In a similar contest, another concept might be found in interventricular desynchrony. During normal cardiac function, contraction and relaxation of both ventricles occurs somehow simultaneously. Ventricular distortion however varies among the patients with PAH, while the RV free wall continues to shorten even after the pulmonary valve closes and the LV free wall begins to relax as soon as the aortic valve closes (31). In summary, aberrant loading of one ventricle impacts the structure and functionality of the other due to the ventricles' series coupling within the pericardium. This ventricular dependency in PAH can be shown by monitoring the distortion of the LV and RV.

## Radiological approach in early diagnosis of ventricular dysfunction in PAH

### Transthoracic echocardiography

**Figure 1:**
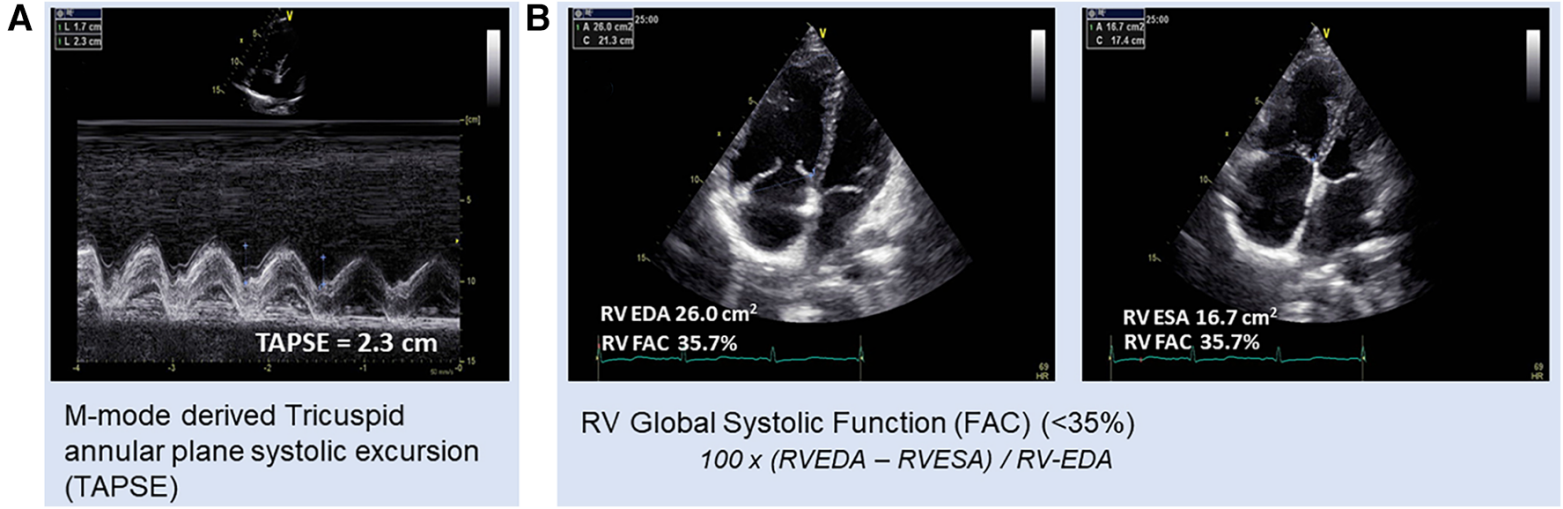
Patient with pulmonary hypertension, (**A**) TAPSE with respiratory fluctuations (2.3 and 1.7, respectively) and (**B**) RVFAC = 35.7%. RV-FAC, right ventricular fractional area change; RV EDA, right ventricular end-diastolic area; RV ESA, right ventricular end-systolic area.

According to the diagnostic algorithm, transthoracic echocardiography (TTE) is the next suggested diagnostic test for individuals with a history and signs or symptoms indicative of PH (32). Based on current guidelines, the pulmonary arterial systolic pressure (PASP) is determined by combining the peak tricuspid regurgitant (TR) velocity with additional two-dimensional (2D) and Doppler findings to assess the likelihood of PAH and determine whether additional evaluation is necessary (7). In addition to the more traditional and well-established assessments of RV function, more recent years have seen the introduction of some highly intriguing non-conventional approaches. Most often, the tricuspid annular plane systolic excursion (TAPSE) is used as a stand-in for the RV function (33). TAPSE (Figure 1A) is a powerful 2DE tool for measuring RV function as well as a significant parameter for disease prognosis (34–36). The significance of RV TAPSE in RV function in patients with PH was first carried out by Forfia in 2006 (34). A cutoff value below 18 mm was associated with RV dysfunction and worse survival. In a study by Vitarilli et al. (37), for a cutoff value of 16 mm, the sensitivity and specificity of RV failure were found to be 76% and 64%, respectively. In the case of TAPSE as well as for right ventricular functional area change (RVFAC), at a cutoff value of 38% the sensitivity and specificity for RV failure were found to be 72% and 60%, respectively. Similarly, in 146 patients with PAH, a study for the prognostic value of echocardiography by Mercurio et al. (38) found that TAPSE and degree of tricuspid regurgitation were highly associated with survival (*p* < 0.01, *p* = 0.04, respectively). Nevertheless, it was unable to predict mortality in later research, particularly in the New York Heart Association (NYHA) Classes III–IV and RV dilatation (39). In addition, it predominantly mirrors the longitudinal RV function and is dependent on volume (39). It has been demonstrated that RVFAC (Figure 1B) and right ventricular size, measured at the end diastole, are predictive of survival (39). RVFAC is a recognized prognostic indicator of heart failure and sudden cardiac death in the course of PAH (40).

**Figure 2:**
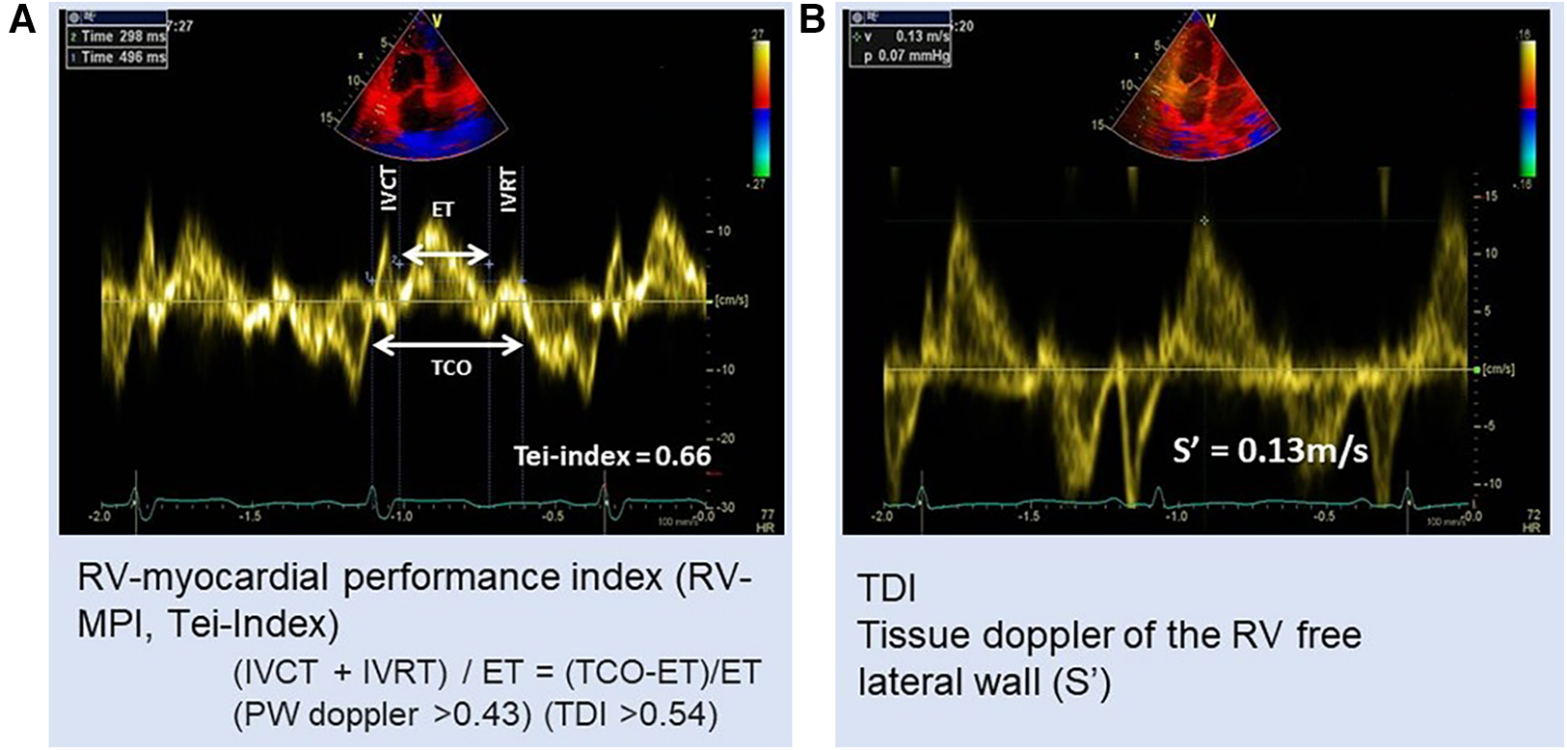
Patient with PH: (**A**) RV myocardial performance index (MPI) or Tei-index = 0.66 and (**B**) tissue Doppler velocity of RV free wall S′ = 0.13 m/s. ET, ejection time; IVCT, isovolumetric contraction time; IVRT, isovolumetric relaxation time; TCO, tricuspid valve closure to opening time.

RV-myocardial performance index (RVMPI) or Tei-index (Figure 2A) is another non-conventional method as a combined indicator of both systolic and diastolic functions. RVMPI for the assessment of RV was first described by Tei in 1996 (41). In their model, the index was increased by 0.93 in PH vs.0.28 in the control group. A higher RVMPI of 0.88 or 0.688 indicated a higher chance of survival (39, 42). A study by Vorhies et al. (43) demonstrated that LV and RVMPI markers of global ventricular function were significantly increased in patients with PH compared with in the normal control. However, RVMPI is a load-dependent parameter that increased afterload and reduced preload increase the index (44). Hence the index could not be used to assess change in ventricular contractile function.

### 2D speckle tracking echocardiography

2D speckle tracking echocardiography (STE) has shown itself to be a dependable, accurate, feasible, and angle-independent quantitative method for assessing RV function in recent years (36, 45). The advantage of this method is that it provides a very precise analysis of individual myocardial segments' functions (46). Myocardial strain (ε) refers to the alteration in cardiac tissue caused by an external force, calculated using the formula ε = (Lsystole—Ldiastole)/Ldiastole, where L represents length, and the result is multiplied by 100 to express myocardial deformation as a percentage throughout the cardiac cycle. A positive value signifies lengthening, while a negative value indicates shortening. Utilizing strain imaging presents a viable non-invasive method for evaluating cardiac mechanics, offering the potential to detect subclinical ventricular dysfunction (45). Strain derived from STE can be delineated across various regions of the RV free wall or presented as an average encompassing visualized segment, recognized as the GLS. The results are expressed as a percentage, where a more negative numerical value denotes greater shortening of the myocardial segment during systole. Deterioration in strain is indicated by a less negative number or lower absolute value than anticipated, indicating reduced deformation along the longitudinal axis. GLS conventionally encompasses the basal, midventricular, and apical RV free segments, but may also extend to encompass the basal, midventricular, and apical segments of the IVS (45). The most common measurement of strain in the RV is GLS; however, individual longitudinal segmental strain is also being investigated in PH (47).

STE permits the early diagnosis of subclinical RV impairment (48). Speckle-tracking echocardiography, employed for ventricular strain and torsion analysis, has augmented the understanding of alterations in systolic function among adult patients with PH. Sachdev et al. (49) demonstrated that LR longitudinal peak systolic strain (−15 ± 5%) and strain rate (−0.80 ± 0.29 s) are predominantly decreased in patients with PAH. Notably, discernible reductions in systolic torsion, as well as longitudinal and circumferential strain, have been demonstrated through this analytical approach (25). Diminished strain emerges as an early indicator of RV dysfunction, as individuals exhibiting decreased longitudinal deformation experience more unfavorable outcomes compared with counterparts with equivalent right heart dimensions and TAPSE (50, 51). The RVGLS shows significant prognostic significance in patients with PAH. A 51 consecutive patients with PAH study by Park et al. (48) showed significant prognostic information in such patients. RVGLS (HR = 2.090, *p* = 0.042) was found to be a significant predictor of death. Impaired RVGLS (≥ −15.5%) was associated with lower event-free survival (HR = 4.906, *p* = 0.001) and increased mortality (HR = 8.842, *p* = 0.005). Similarly, in another 45 prospective consecutive patients with PH study by Fukuda et al. (52) with RV free wall longitudinal strain and RV septal wall longitudinal strain, the RV-free wall longitudinal strain by multivariate analysis revealed that the RV free wall was an independent echocardiographic predictor of hemodynamic RV performance items and RV ejection fraction well correlated with cardiac magnetic resonance and found the potential role for follow-up in patients with PH. Badagliacca et al. (53) showed that even when standard parameters like RV-FAC were included in the multivariate analysis, RV desynchrony evaluated with 2Decho-strain had the strongest predictive capability of peak V'O2. However, 2D STE only measures the deformation in the 2D plane and thus the deformation in other directions is not feasible to assess. Furthermore, 2D STE assessment is only available in the apical four-chamber view precluding the evaluation of the outflow portion of the RV. Mukherjee et al. (50) aimed to investigate the clinical usefulness of STE to distinguish differential RV contractility between PAH subtypes (50). In a cohort of 55 patients with PAH (32 SSc-PAH and 23 IPAH), the authors found significant regional differences in RV function between SSc-PAH and IPAH at similar afterloads. Notably, although while patients with SSc-PAH had shorter durations of PAH illness, they still had anomalies in RV contractile as identified by RVLSS. However, results point to differential RV myocardial illness that separates non-invasively detectable PAH subtypes that may be linked to SSc-specific microvascular disease and/or myocardial fibrosis (50).

As already stated, due to the continuous circulatory system and shared septum, the RV–LV interdependence is a great matter of concern. Researchers have claimed that through ventricular dependence there is LV dysfunction in PH (26). A study by Burkett et al. (54) found that LVGLS was found significantly reduced in patients with PH in comparison with matched controls. The authors also stated that the strain and strain rate were predominantly found reduced within the septum in relationship with invasive hemodynamics and functional PH measures (54).

### Tissue Doppler imaging

RV function can also be evaluated by measuring the longitudinal peak velocity at the basal segment of the free wall using tissue Doppler imaging (TDI), and tissue velocities change during the isovolumic phase (55). There is an entire review article based on Doppler measurements of RV function (56). In PH and associated right ventricular remodeling due to increased afterload, functional tricuspid regurgitation takes place (57). Tricuspid regurgitant velocity (TRV) is still a classic measurement for screening purposes of patients with suspected PH (57). The tricuspid regurgitant pressure gradient (TRPG) can be calculated from TRV using Bernoulli equation, (*P* = 4 V^2^). A study by Fisher at al. validated the accuracy of TRV against catheter, and inaccuracy was found greater than 48 mmHg in 48% cases (58). In the absence of pulmonary valve stenosis, the resultant pressure is equivalent to pulmonary arterial systolic pressure. While, this aids in the assessment of pulmonary pressure, it does not reflect RV function (58). In conclusion, TRV is angle dependent and depends on the right heart function. Right HF may result in less TRV because of reduced contraction and greater annular dilatation.

Tricuspid annular systolic (S′) velocity (Figure 2B) and S′ velocity time integral (VTI) are other tissue Doppler measurements. S′ and VTI measurements were found significantly decreased in pulmonary hypertensive patients in comparison with control and were found consistent with RV systolic dysfunction (43). For the assessment of RV function, a value of less than 10 cm/s is now recommended as a measure of RV impairment in echocardiographic guidelines (59). TDI also allows assessment of LV diastolic function by direct measurements of myocardial velocities independently of cardiac preload (24).

### 3D echocardiography

**Figure 3:**
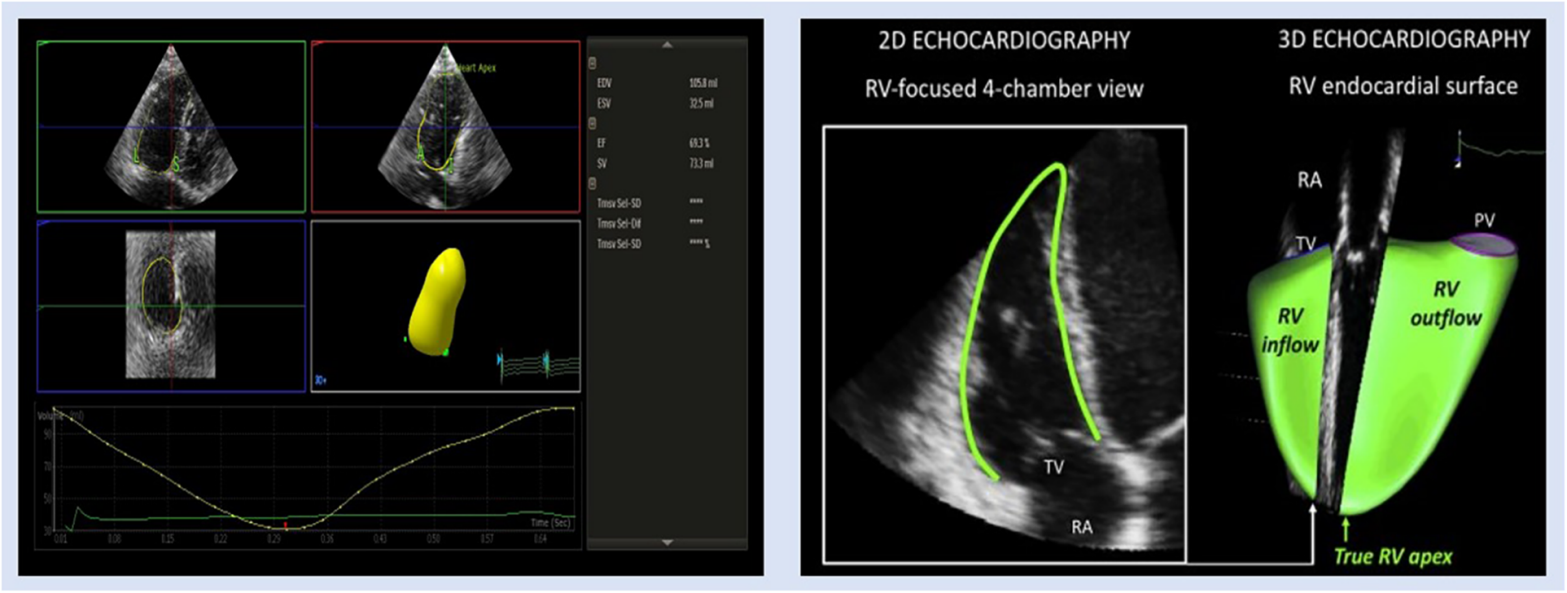
Representative images of 3DE RV assessment in patient with PH. EDV, end-diastolic volume; ESV, end-systolic volume; EF, ejection fraction; RA, right atrium; PV, pulmonic valve; SV, stroke volume; TV, tricuspid valve.

Owing to the complex geometry, crescent shape, and complex mode of contractility of the RV, 2D echocardiography (2DE) is unable to locate the RV inlet and outlet in the same image. Consequently, three-dimensional echocardiography (3DE) is a more precise and feasible tool than 2DE for the assessment of RV volumes and function (60). RV function measured with 3DE (Figure 3) was found to be more closely correlated with gold standards CMR and cardiac catheterization (36, 61–63). Despite this, the accessibility of 3D echo is greater than CMR, which makes this an attractive alternative. In addition, strain imaging has been combined with 3DE to accurately predict RV ejection fraction (EF) (64). A prior investigation by Vitarelli et al. (37) revealed a moderate association (Pearson correlation coefficient ranging from −0.51 to −0.64) between 3DE-RVEF and 3D-RVEF exhibited the highest area under the curve (0.89, *P* < 0.05) when compared with other 3DE parameters for hemodynamic indices found by RHC in identifying hemodynamic indicators of RV failure (a composite of Ci < 2 L/min/m^2^ and RA *P* > 15 mmHg). By receiver operating curve (ROC) analysis, 3DE-RVEF (0.89) has the highest area under the curve (AUC) (95% CI; 0.68–0.99, *P* < 0.05) with the cutoff value of EF 39% and 90% and 83% sensitivity and specificity, respectively. The most recent study by Liu et al. (63) demonstrated that 3DE-derived RVEF had the potential to predict the intermediate–high risk stratification (AUC 0.82, 95% CI 0.73–0.91, *P* < 0.001), which showed the best predictive capacity. To be specific, EF < 26.39% had an 81.6% sensibility and 73.8% specificity to predict intermediate–high risk stratification. 3D-RVEF (OR 0.82, 95% CI 0.75–0.90, *P* < 0.001) was identified as an independent predictor of intermediate–high risk stratification in patients with PAH. The authors concluded that 3DE-derived EF and free wall strain (FWS) had the potential to independently predict intermediate–high risk stratification in patients with PAH, and the predictive capacity was retained after adjustment for demographic data. Similar findings were found by Murata et al. (65), whose study's ROC analysis showed that 3DE-RVEF was more accurate in predicting cardiac events, such as hospitalization, intervention, and mortality than other hemodynamic measurements, such as mPAP, with an AUC of 0.78.

3DE-derived speckle tracking has proved itself a reliable method for RV systolic function evaluation and survival prediction (61, 64). Smith et al. (64) aimed to assess the RV strain with 97 consecutive patients with established pulmonary hypertension disease in comparison with 60 controls, with the help of 3DE-derived strain. In comparison with normal controls, the strain analyses (area strain, redial strain, circumferential strain, and longitudinal strain) were all found reduced. RV global area strain (RVGAS) was found to be a significant determinant of all-cause mortality [HR: 3.49; 1.21–7.07 with 95% confidence interval (CI); *P* = 0.017]. Hence, 3DE-derived strain analysis is a reliable tool for the assessment of disease outcome in such patients.

### Cardiac magnetic resonance imaging

A non-invasive method for obtaining detailed, three-dimensional pictures of the heart is cardiac magnetic resonance imaging (CMRI). It offers details on the anatomy, physiology, and structure of the right heart that are not easily accessible by other techniques like echocardiography and RHC. The most reliable method for measuring RV volume and ejection fraction (RVEF) is CMR, especially when steady-state free precession (SSFP) cine imaging is employed (66). A study by van de Veerdonk et al. (67) suggested that RVEF measured by CMRI was a better predictor of mortality than PVR (area under the ROC curve: 0.749 vs. 0.628). A sample of patients with IPAH was researched by Holverda et al. (68) to determine how their stroke volume responded to exercise. MRI was used to evaluate cardiac function both at rest and during submaximal activity. They discovered that patients with IPAH had a decrease in RVEF during exercise, indicating a symptom of RV failure.

**Figure 4:**
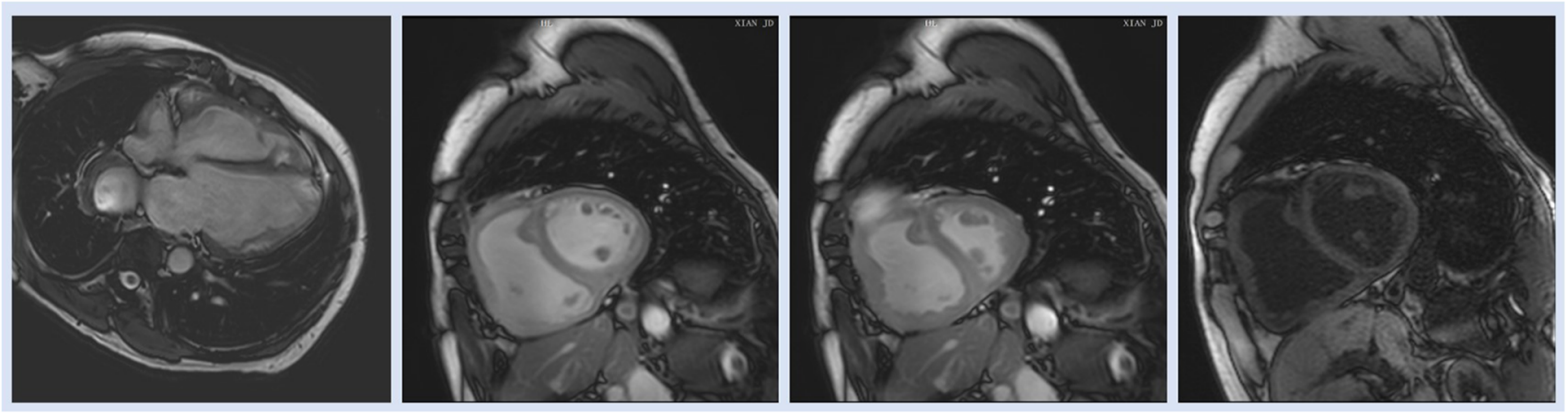
Representative images of cardiac magnetic resonance imaging in patient with PH.

T1 mapping, a newly developed CMRI technique, measures native RV T1 based on pre- and postcontrast T1 timings to detect widespread myocardial anomalies (69). T1 mapping may be a useful tool for identifying fibrosis, which has recently been linked to both maladaptive responses that lead to increased diastolic stiffness and adaptive responses that prevent cardiomyocyte overstretch and preserve RV shape for optimal function (70). Similarly, in a case control study Ostenfeld et al. (17) demonstrated that despite a preserved global LV function, the LV function is affected in patients with PH. They found that LV atrioventricular plane displacement (AVPD) and corresponding longitudinal contribution to LV SV was lower in patients compared with controls. Interestingly, the decreased longitudinal contribution to LVSV was not seen in the RV, contrary to previous findings in RV volume overload (17) (Figure 4).

### CMR tissue-tracking technology

Tissue-tracking (TT) technology (strain analysis) has been used in recent years to get myocardial strain parameters by postprocessing typical CMRI data; this method does not require additional sequences or pictures (71, 72). Evaluating RV myocardial deformation can identify subclinical cardiac failure because a recent study found that there is variation in local RV function in patients with PH before a drop in the RVRVEF is evident (71). A recent study by Cao et al. (73) aimed to evaluate both left and right ventricular functions in patients with PAH by using cardiac magnetic resonance tissue tracking technology (CMR-TT) to explore its clinical value. A total of 79 participants (47 patients with PAH and 32 healthy controls) underwent CMR with a steady-state free precession (SSFP) sequence. The results showed that global radial strain (GRS), global circumferential strain (GCS), and global longitudinal strain (GLS) were significantly found lower in patients with PAH in comparison with the healthy controls (73). The authors concluded that CMR-TT is a non-radioactive and non-invasive powerful tool for evaluating RV and LV dysfunction in PAH with preferable feasibility and repeatability (73).

### Nuclear medicine

The first-pass radionuclide angiography (FP-RNA) method is regarded as a gold standard method for RVEF measurement. Imaging is obtained dynamically after the first quick transit of administered radiolabeled red blood cell (RBC) over a brief period (5–10 cardiac cycles) via the right atrium, right ventricle, pulmonary artery, lungs, and left heart. Because overlapping activity from overlaid structures is eliminated, FP-RNA can reliably quantify RVEF as the radioactive bolus moves through the heart chambers and major arteries (74). However, because of its technological complexity, this approach depends on a high-quality tracer bolus injection, quick imaging capture, and the right postimaging data processing and reconstruction. An alternative method for calculating RVEF is the Gated Blood Pool SPECT (GBPS) instead of FP-RNA. GBPS involves a tomographic acquisition of the heart, allowing for the separation of the RV from the right atrium and LV. This characteristic makes GBPS particularly well-suited for precise RVEF measurements. Furthermore, the imaging acquisition process for GBPS is simpler and boasts a higher success rate compared with FP-RNA (74). RVEF calculations in patients with chronic thromboembolic pulmonary hypertension were performed using both GBPS and FP-RNA. The results indicated a strong correlation between the two methods (*r* = 0.68, *P* < 0.0001), but the RVEF calculated using GBPS was also overestimated (51% + 14% calculated from GBPS vs. 37% + 12% from FP-RNA, *P* < 0.01) (75). In conclusion, RVEF should be measured using FP-RNA if methodology is not an issue. Otherwise, because of its greater procedural success rate and relatively simple imaging capture, GBPS is advised. RVEF is computed using correlations from GBPS. This tool is not used routinely for RVEF measurements because it does not provide other parameters such as RV volume as it is purely counting base calculation.

Sympathetic nervous system (SNS) hyperactivity has a well-recognized role in the pathophysiology of HF with reduced LVEF. Changes in the sympathetic nervous system have been linked to the PAH pathogenesis. The cardiac SNS activity in 12 consecutive patients with PAH was investigated to find a potential relationships with disease severity indicators using ^123^I-metaiodobenzylguanidine (^123^I-MIBG) nuclear imaging, (76). When measured as the early and late heart-to-mediastinum ratio (H/M), cardiac ^123^I-MIBG uptake in PAH was considerably lower than in controls. The study showed significant impairment in cardiac SNS in PAH. This impairment correlated with indices of PAH severity (76). Cardiac sympathetic dysfunction may be a contributing factor to the development of right heart failure in PAH.

### PET scanning

PAH is typified by perivascular inflammation, smooth muscle layer hypertrophy, lung vascular intimal lesions, and concurrent RV remodeling. These factors may eventually result in a reduction in RV function such as RV failure. RV failure development is largely determined by the RV metabolic state, which is defined by the degree of ischemia and glycolysis. These factors combine to form a vicious cycle that includes hypoxia, increased RV systolic pressure (RVSP), decreased RV output, and additional myocardial ischemia. In this contest, 2-deoxy-2-[(18)F]fluoro-D-glucose (FDG) positron emission tomography (PET) has been used for the measurement of glucose uptake (GU) as an indicator of glucose metabolism in the right heart and pulmonary vasculature (77). Currently, non-invasive imaging of the size and function of the RV is used in the clinical evaluation of patients with PAH, and the 6-min walk test and cardiopulmonary exercise testing (CPET) are used to measure exercise capacity. Invasive hemodynamic evaluation is also performed on these patients. Nonetheless, patients with PAH exhibit a great deal of diversity in their clinical course. Studies suggest that increased pulmonary arterial pressures are associated with higher 18F-fluorodeoxyglucose (FDG) uptake in the right ventricle using PET imaging (78). There is proof that pressure overload causes a “metabolic shift” in cardiomyocytes, and FDG uptake is correlated with the advancement of PAH and unfavorable outcomes (79). A study by Bukhari et al. (80) compared the two ventricles to determine the RV and LV indices determined by PET scanning to correlate with the severity of IPAH as determined by hemodynamic and CPET; the study assessed myocardial blood flow (MBF) and substrate metabolism for the RV and LV in IPAH. The findings of this investigation shed light on how increases in RV afterload affect RV blood flow and substrate metabolism. They also showed that RV blood flow and metabolism in patients with IPAH can be measured using dynamic PET scanning. They concluded that in patients with IPAH, PET scanning using 13N-NH3 and ^18^F-FDG seems to be a practical method for measuring RV blood flow and RV metabolism.

Another concept in PET scanning is the integrated hybrid design, which is hybrid PET/MRI scans in patients diagnosed with PAH. With benefits for imaging dynamic processes visible on either PET or MRI, the integrated hybrid design offers genuinely simultaneous PET and MRI (81, 82). This new non-invasive diagnostic tool is the simultaneous PET/MRI system. PET is a cutting-edge technique that employs short-acting radiopharmaceuticals to enable molecular imaging and provide information on the intracellular metabolic processes occurring within cells. MRI, the second part of this hybrid system, produces very high-quality anatomical images (83). To sum up, elevated glucose metabolism in the RV as shown by FDG PET is linked to progressive alterations in the pulmonary vascular bed and reduced RV function. When PET and MRI are combined, it may be possible to provide important information about RV function and identify clinical worsening early in patients with PAH (83).

### Challenges and future directions

Non-invasive imaging modalities play a crucial role in the early diagnosis of right and left ventricular functions in PH. Echocardiography is often the initial choice, offering a real-time assessment of pulmonary pressures, RV size, and function, and the presence of tricuspid regurgitation. However, echo has limitations in fully characterizing RV function and may not capture subtle changes. CMR emerges as a powerful tool, providing detailed anatomical and functional information, including accurate RV volumes, function, mass, and myocardial strain. CMR is particularly adept at identifying early structural changes in both ventricles, contributing valuable insights into PH progression. PET scans, with their ability to assess metabolic and cellular activity, offer additional perspectives on myocardial function in PH, aiding in early detection. Challenges in these modalities include limited availability, cost considerations, and the need for specialized expertise as well as some sort of radiation exposure. Future directions involve the refinement of quantitative parameters for early PH detection, the integration of artificial intelligence to enhance accuracy, and the development of standardized protocols to facilitate multicenter research. Combining the strengths of echo, CMR, and PET holds promise in advancing our understanding of PH pathophysiology, facilitating early diagnosis, and tailoring targeted interventions for improved patient outcomes. Techniques such as advanced strain imaging, four-dimensional (4D) flow MRI, and parametric mapping in cardiac MRI may provide more sensitive indicators of early ventricular dysfunction in PH. Artificial intelligence (AI) and machine learning algorithms (MLA) have the potential to revolutionize the interpretation of imaging data. These technologies can aid in the automated analysis of complex datasets, enabling quicker and more accurate identification of subtle changes in ventricular function associated with PH.

Similarly, integrating imaging findings with circulating biomarkers associated with early ventricular dysfunction may provide a more comprehensive diagnostic approach. Combining imaging and biomarker data could enhance the sensitivity and specificity of early PH detection. While these advancements hold great promise, their successful integration into clinical practice will require rigorous validation, standardization, and collaboration among researchers, clinicians, and industry stakeholders. Continuous innovation in radiological imaging technologies has the potential to significantly improve the early detection and management of left and right ventricular dysfunction in pulmonary hypertension, ultimately leading to better patient outcomes. In addition, as described earlier about the RV–LV interdependence concept, the LV dysfunction in PAH is a hot topic to explore and discus further.

## Discussion

This article highlights various non-invasive imaging modalities used for the early detection and assessment of left and right ventricular dysfunction in PH and the prognostic utility of each approach (Tables 2, 3). Each modality has its own strengths and limitations, which are discussed in Table 4 in detail. Conventional 2D TTE, speckle-tracking strain imaging, and 3D echocardiography are more specialized techniques that are increasingly becoming part of the standard of care in monitoring right heart structure and function. The 2D traditional parameters such as tricuspid regurgitant velocity and TAPSE for RV function assessment play a crucial role. However, the limitations of TAPSE in predicting mortality and its dependence on volume are noted. Advanced techniques such as 2D STE provide more comprehensive insights into RV function, with the RVGLS demonstrating significant prognostic significance in PH patients. TDI and 3DE offer additional perspectives, particularly in evaluating longitudinal peak velocity and RV volumes, respectively.

**Table 2:** Modalities hazardous analysis for mortality in patients with PAH using different approaches.

Modalities	Author	*n*	Approach	Follow-up (month)	Cutoff value	HR	95% CI	*P*-value
2DE	Forfia et al. (34)	47	TAPSE (Continuous)	19.3	<18 mm	1.17	1.04–1.32	<.001
		47	TAPSE (Dichotomous)	24	<18 mm	5.69	1.30–24.95	0.01
	Park et al. (48)	51	TAPSE^b^	45	<15 mm	0.64	0.329–1.245	0.189
	Moceri et al. (62)	104	TAPSE	6.7	16 mm	0.72	0.52–0.99	0.04
	Smith et al. (64)	97	TAPSE	24	<18 mm	2.12	0.89–5.34	0.09
	Jone et al. (36)	96	TAPSE^b^	24	NA	0.82	0.32–2.10	0.068
	Vitarelli et al. (84)	66	TAPSE	6	13 mm	1.74	1.53–2.86	<.05
	Grapsa et al. (39)	572	TAPSE^b^	84	<15 mm	0.95	0.91–0.99	<.001
	Vitarelli et al. (37)	73	RVFAC	42	38%	NA	0.47–0.86	0.08
	Forfia et al. (34)	47	RVFAC	19.3	NA	0.92^a^	0.86–0.96	0.02
	Vitarelli et al. (84)	66	RVFAC	6	37%	1.98	1.63–2.95	<.01
	Grapsa et al. (39)	572	RVFAC^b^	84	NA	0.97	0.93–0.99	0.038
	Jone et al. (36)	96	RVFAC^b^	24	32%	0.08	0.03–0.22	<.001
	Kishiki et al. (19)	114	RVFAC^b^	20	NA	0.64	0.91–0.97	<.01
	Park et al. (48)	51	RVFAC^b^	45	NA	0.636	0.358–1.130	0.123
	Grapsa et al. (39)	572	RVMPI^b^	84	≥0.64	4.47	1.11–17.94	0.35
	Jone et al. (36)	96	RVGLS^b^	24	16%	0.17	0.07–0.45	<0.001
	Kishiki et al. (19)	114	RVGLS^b^	20	NA	1.08	1.01–1.15	0.03
	Vitarelli et al. (84)	66	RVGLS	6	−19%	2.27	1.49–3.92	<.0005
		66	LVGLS	6	NA	1.11	1.01–1.22	0.04
	Moceri et al. (62)	104	RVGAS	6.7	−18%	1.72	1.07–2.78	0.03
	Park et al. (48)	51	RVGLS	45	−15.5%	2.09	1.054–4.145	0.042
3DE	Vitarelli et al. (37)	73	RVGLS	42	−17%	4.6	2.79–8.38	0.004
	Smith et al. (64)	97	RVGLS	24	>−16.1%	7.63	1.76–10.27	0.112
	Smith et al. (64)	97	RVGAS	24	>−24.7%	3.49	1.21–7.07	0.017
	Smith et al. (64)	97	RVEF	24	<30.3%	2.43	1.00–5.92	0.05
	Moceri et al. (62)	104	RVEF	6.7	28%	0.8	0.64–0.99	NA
	Vitarelli et al. (84)	66	RVEF	6	40%	3.51	1.66–4.51	0.001
	Jone et al. (36)	96	RVEF^b^	24	43%	0.1	0.03–0.27	<0.0001
	Vitarelli et al. (37)	73	RVEF	42	39%	5.3	2.85–9.89	0.002
CMR	Zhong et al. (72)	169	PAGLS	34	9%	2.93	NA	0.01
	van de Veerdonk et al. (67)	76	RVEF	12	35%	0.92	0.875–0.985	0.014
		76	LVEF	12	35%	0.98	0.947–1.031	0.576
PET/MRI	Kazimierczyk et al. (83)	26	RVEF	14.2	42%	0.95	0.88–1.04	0.3
		26	LVEF	14.2	NA	1.05	0.96–1.11	0.23

^a^ Unadjusted HR.

^b^ Univariable analysis.

2DE, two-dimensional echocardiography; 3DE, three-dimensional echocardiography; CI, confidence interval; CMR, cardiac magnetic resonance; HR, hazard ratio; PET/MRI, positron emission tomography/magnetic resonance imaging; RVAS, right ventricular area strain; RVEF, right ventricular ejection fraction; RVGAS, right ventricular global area strain; RVGLS, right ventricular global longitudinal strain; RVEF, right ventricular ejection fraction; RVFAC, right ventricular fractional area change; TAPSE, tricuspid annular plane systolic excursion; NA, not available; *n*, number of patients.

**Table 3:** Comparative evaluation of parametric approaches in PAH for accurate hemodynamic risk stratification.

Modalities	Authors	*n*	Approach	Follow-up (month)	Cutoff value	AUC	95% CI	% Sensitivity	% Specificity	*P* value
2DE	Vitarilli et al. (37)	73	TAPSE	24	16 mm	0.67	0.53–0.88	76	64	0.05
	Forfia et al. (34)	47	TAPSE	12	<18	0.87	0.77–0.96	NA	NA	<.0,001
	Vitarelli et al. (84)	66	TAPSE	6	13 mm	0.70	0.51–0.96	72	65	0.07
	Moceri et al. (62)	104	TAPSE	6.7	16 mm	0.81	0.72–0.88	68	86	<0.001
	Smith et al. (64)	97	TAPSE	24	18 mm	0.63	NA	70	59.4	NA
	Jone et al. (36)	96	TAPSE	24	14 mm	0.68	NA	60	57	NA
	Vitarilli et al. (37)	73	RVFAC	24	38%	0.62	0.47–0.86	72	60	0.08
	Jone et al. (36)	96	RVFAC	24	32%	0.80	NA	97	57	NA
	Vitarelli et al. (84)	66	RVFAC	6	37%	0.71	0.52–0.96	73	64	0.08
	Vitarilli et al. (37)	73	RVGLS	24	−18%	0.88	0.69–0.98	89	74	0.01
	Park et al. (48)	51	RVGLS	45	≥ −15.5%	0.73	0.592–0.848	70	77	0.044
	Vitarelli et al. (84)	66	RVGLS	6	−19%	0.77	0.63–0.82	77	67	0.06
3DE	Vitarilli et al. (37)	73	RVGLS	24	−17%	0.88	0.71–0.97	89	77	0.01
	Smith et al. (64)	97	RVAS	24	−24.7%	0.67	NA	80	35.5	NA
	Moceri et al. (62)	104	RVGAS	6.7	>−18%	0.83	0.74–0.90	94	62	<.001
	Smith et al. (64)	97	RVGLS	24	−16.1%	0.70	NA	90	52.1	NA
	Liu et al. (63)	91	RVEF	12	<26.39	0.82	0.73–0.91	81.6	73.8	<0.001
	Smith et al. (64)	97	RVEF	24	30.30%	0.61	NA	65	59.2	NA
	Vitarilli et al. (37)	73	RVEF	24	39%	0.89	0.68–0.99	90	83	<0.01
	Moceri et al. (62)	104	RVEF	6.7	NA	0.80	0.71–0.87	75	85	<0.001
	Jone et al. (36)	96	RVEF	24	43%	0.83	NA	90	67	<.0,001
	Vitarilli et al. (84)	66	RVEF	6	40%	0.89	0.72–0.98	88	79	0.02
PET/CMR	Kazimierczyk et al. (83)	26	RVEF	14.2	42	0.76	0.56–0.96	NA	NA	NA

2DE, two-dimensional echocardiography; 3DE, three-dimensional echocardiography; AUC, area under the curve; CI, confidence interval; RVAS, right ventricular area strain; RVEF, right ventricular ejection fraction; RVGAS, right ventricular global area strain; RVGLS, right ventricular global longitudinal strain; RVEF, right ventricular ejection fraction; RVFAC, right ventricular fractional area change; TAPSE, tricuspid annular plane systolic excursion; NA, not available; *n*, number of patients.

**Table 4:** Summary of imaging techniques for RV functional assessment in PAH.

Modalities	Parameters	Recommended methods	Strengths	Limitations	Predictive value
Assessment of RV Function					
Two-dimensional echocardiography (2DE)	TAPSE	Apical 4CV	• Validated against radionuclide EF. • Used as a stand-in for RV function.	• Only shows the longitudinal RV function. • Volume dependent. • Predominantly mirror longitudinal RV function and not applicable in case of regional RV function. • Angle dependent.	Low TAPSE is associated with lower CI and worse survival (34, 35).
		Maximum systolic excursion of the lateral tricuspid annulus (M-mode) between end-diastole and peak systole (<16 mm).			
	TDI	The longitudinal velocity of the tricuspid annular plane (S′ value <0.095 m/s)	• Reproducible • Validated against radionuclide EF. • Good correlation with normal control	• Angle dependent. • Requires a high frame rate. • Reflect the function of a small subsection of the RV, (Basal) section. • Cannot apply to regional wall motion abnormalities.	Established Prognostic Value in PAH (34).
	Tissue Doppler of the RV free lateral wall (S′)				
	Global longitudinal strain (GLS),	% systolic shortening of the RV free wall.	• Regional and global function. • Sensitive tool for identifying initial RV dysfunction. • Angle independent. • Highly reproducible. • Provide information about the whole myocardium thickness. • Less load dependent. • Well correlated with RV SVI, RA pressure, and RV function.	• Vendor and software dependent. • Dependent on RV size and Shape. • Dependent on image quality. • Load dependent (Less than PW and TDI). • Strain rate velocity is less accurate than time-integrated measures such as strain. • RV free wall GLS corresponds better with RV mechanical function than RV global strain (which includes IVS and is part of both ventricles). • In patients with AF average of three heart cycle.	Established prognostic significance in PAH (49, 85).
	RV Free wall strain.	Average peak systolic strain of the three segments of the free lateral wall.			
	RV Global Systolic Function (FAC) (<35%)	100x (RV-EDA—RV ESA)/RV-EDA	• Good correlation with CMR-derived RV function. • Reflects both radial and longitudinal contraction.	• Dependent on image quality. • Delineation between endocardium and trabeculations. • Missing the contribution of RVOT to overall RV systolic function.	Established predictive value with good sensitivity and specificity (34).
		RV focused 4CV			
	RV myocardial performance index (RVMPI, Tei Index)	Tissue Doppler velocities or pulse wave velocities from the RV (IVRT + IVCT)/RV ejection time (PW Doppler >0.43) (TDI >0.54)	• Reflects global RV function. • Do not require full visualization of the RV.	• Affected by irregular heart rate. • Elevated RA pressure decreased IVRT.	The established survival rate with good RVMPI (86).
Three-dimensional echocardiography (3DE)	3DE-derived RVEF	RV focused A4C.	• Reliable. • Reproducible. • Good correlation with the gold standard (CMR). • Includes RVOT function to overall function.	• Dependent on image quality. • Load dependent. • Not applicable in patients with arrhythmia. • Requires great expertise and offline analysis.	Good correlation with RHC as a hemodynamic indicator.
		RF EF (%) = 100x(EDV-ESV)/EDV (RVEF <45%)			
Cardiac magnetic resonance (CMR)	CMR-derived RVEF	Steady-state free precession (SSFP) dynamic gradient-echo cine loop using retrospective ECG gating and parallel imaging technique with a 6-mm slice thickness of the imaging plane	• Gold standard. • Non-invasive.	• Common contraindicated cardiac magnetic devices such as left ventricular assist devices. • Claustrophobia. • Time consuming and relatively expensive.	Established predictive value (67).
	CMR Tissue-tracking technology (Strain analysis)	CMR with steady-state free precession (SSFP) sequence	• Non-radioactive. • Non-invasive. • Preferable feasibility and repeatability. • Not require additional sequences or pictures.	• Lower spatial and temporal resolution. • Operator dependence. • Cost-effective.	
Nuclear medicine	First-pass radionuclide angiography (FP-RNA)	Dynamic initial rapid transit of injected radiolabeled RBC through the RV, RA, PA, lungs, and left heart during a short time interval (5–10 cardiac cycles).	• Gold Standard. • Preferable feasibility and repeatability. • Can assess bi-ventricular function.	• Semi-invasive in nature. • Requires technically demanding procedure such as, • High quality of tracer bolus injection. • Prompt imaging acquisition. • Appropriate postprocessing imaging data.	Prognostic value unknown.
Positron emission tomography (PET)			• Provide functional information about the tissue. • Can detect abnormalities at the cellular level. • Multi-Tracer Imaging.	• Limited spatial and temporal resolution. • Radiation exposure. • Cost-effective. • Dependency on Tracers.	Established prognostic value (83).

4CV, four chamber view; AF, atrial fibrillation; CMR, cardiac magnetic resonance; ECG, electrocardiogram; EDV, end-diastolic volume; EF, ejection fraction; ESV, end-systolic volume; FAC, fractional area change; IVCT, isovolumetric contraction time; IVRT, isovolumetric relaxation time; PW, pulsed wave; RA, Right atrium; RHC, right heart catheterization; RV, right ventricle; RVEF, right ventricular ejection fraction; RVEDd, right ventricular end-diastolic diameter; RVESd, right ventricular end-systolic diameter; RVMPI, right ventricular myocardial performance index; RVOT, right ventricular outflow tract; TAPSE, tricuspid annular plane systolic excursion; CI, cardiac index; TDI, tissue Doppler imaging; PA, pulmonary artery; PAH, pulmonary arterial hypertension; SVI, stroke volume index.

CMR emerges as a powerful tool, offering precise measurements of RV volumes and ejection fraction. T1 mapping in CMR enables the identification of fibrosis, providing insights into myocardial anomalies associated with PH. CMR tissue-tracking technology, based on strain analysis, proves effective in evaluating RV dysfunction non-invasively. PET scanning, specifically 2-deoxy-2-[(18)F]fluoro-D-glucose (FDG) PET, sheds light on the metabolic state of the right heart and pulmonary vasculature in PAH. The integration of PET/MRI offers a novel hybrid imaging approach, combining molecular and anatomical information for a more comprehensive assessment of RV function.

In synthesis, according to significant discoveries in the recent literature, although PH is predominantly a right heart disease, due to the factor of shared septum and pericardial sac, LV function is also a matter of concern in such patients. Each non-invasive modality mentioned previously offers distinctive contributions to the prompt diagnosis of ventricular dysfunction, thereby facilitating improved timely management in affected individuals. Among these modalities, CMR stands out as a gold standard test for early identification of ventricular function, also serving as a crucial prognostic indicator in such cases. However, it is important to note that CMR does present notable contraindications, including but not limited to claustrophobia, presence of implanted magnetic devices such as left ventricular assist devices, and its high cost. These limitations warrant consideration in routine clinical practice. Recent advancements in echocardiography especially the fully automated 3DE quantification also has an emerging role in patients with PH. Overall, these imaging modalities contribute valuable diagnostic and prognostic information in the context of PH, with ongoing research focusing on refining and expanding these techniques. The exploration of novel biomarkers, artificial intelligence integration, and advancements in imaging technology are anticipated to further enhance the early detection and monitoring of ventricular dysfunction in pulmonary hypertension.

## Conclusion

Non-invasive modalities such as echocardiography, nuclear imaging, and CMR are all useful for screening, classifying, prognosticating, and monitoring the effectiveness of therapy in PH. The standardized algorithm using echocardiographic parameters such as TAPSE, FAC, TDI, and strain analysis in the early diagnosis of dysfunction can also be supplemented with CMR methods. CMR is already considered the gold standard for assessment of the function. Each modality can complement the other in the early diagnosis of ventricular dysfunction in PAH. A thoughtfully crafted clinical strategy should consider the availability of expertise, necessary imaging equipment, and analytic software within a value-based framework. Innovative imaging methodologies like strain imaging, 3D echocardiography, FDG-PET, and dual PET/MRI scan can assess the function of PAH. These techniques can be employed alongside conventional imaging modalities to track disease progression and evaluate the response to therapeutic interventions.
